# Important patient characteristics differ prior to total knee arthroplasty and total hip arthroplasty between Switzerland and the United States

**DOI:** 10.1186/s12891-016-1372-5

**Published:** 2017-01-11

**Authors:** Patricia D. Franklin, Hermes Miozzari, Panayiotis Christofilopoulos, Pierre Hoffmeyer, David C. Ayers, Anne Lübbeke

**Affiliations:** 1 Department of Orthopedics and Physical Rehabilitation, University of Massachusetts Medical School, 55 Lake Avenue North, Worcester, MA 01655 USA; 2 Division of Orthopaedics and Trauma Surgery, Geneva University Hospitals and Geneva University, Geneva, Switzerland

## Abstract

**Background:**

Outcomes after total knee (TKA) and hip (THA) arthroplasty are often generalized internationally. Patient-dependent factors and preoperative symptom levels may differ across countries. We compared preoperative patient and clinical characteristics from two large cohorts, one in Switzerland, the other in the US.

**Methods:**

Patient characteristics were collected prospectively on all elective primary TKAs and THAs performed at a large Swiss hospital and in a US national sample. Data included age, sex, education level, BMI, diagnosis, medical co-morbidities, PROMs (WOMAC pain/function), global health (SF-12).

**Results:**

Six thousand six hundred eighty primary TKAs (US) and 823 TKAs (Swiss) were evaluated. US vs. Switzerland TKA patients were younger (mean age 67 vs. 72 years.), more obese (BMI ≥30 55% vs. 43%), had higher levels of education, more cardiac disease. Swiss patients had lower preoperative WOMAC pain scores (41 vs. 52) but pre-operative physical disability were comparable. 4,647 primary THAs (US) and 1,023 THAs (Swiss) were evaluated. US vs. Switzerland patients were younger (65 vs. 68 years.), more obese (BMI ≥30: 38% vs. 24%), had higher levels of education, more diabetes. Swiss patients had lower preoperative WOMAC pain scores (40 vs. 48 points). Physical disability was reported comparable, but Swiss patients indicated lower mental health scores.

**Conclusion:**

We found substantial differences between US and Swiss cohorts in pre-operative patient characteristics and pain levels, which has potentially important implications for cross-cultural comparison of TKA/THA outcomes. Reports from national registries lack detailed patient information while these data suggest the need for adequate risk adjustment of patient factors.

## Background

Osteoarthritis (OA) of the knee and hip are the most rapidly rising musculosketetal conditions among adults as reflected in the increasing demand for total knee and hip arthroplasties (TKA and THA, respectively) [1, 2]. In the future the prevalence of OA is expected to rise even further due to increases in life expectancy, aging populations, and obesity [3]. OA places a high financial burden on the health care system through costs for medical and surgical treatment, adaptive aids and devices, and time off from work [4]. Reducing OA incidence and progression and improving its treatment are considered urgent public health priorities [5].

Total joint arthroplasty (TJA) is currently the most effective treatment of endstage hip and knee OA. Because surgeons use similar implants internationally, the outcomes of TKA and THA, including patient-reported outcome measures (PROMs), complication rates, and implant survival, are often generalized across countries. Existing research has shown that these outcomes are in part dependent on the patient’s preoperative characteristics [6–14]. However, patient dependent factors can differ considerably between countries and continents, possibly leading to differences in results and national revision burden. National TJA outcome registries often lack detailed patient information, especially BMI, co-morbidities, socio-economic status, pain, and level of function. In addition, few studies have compared these variables between different countries [7, 15–18]. Most of these papers focused primarily on variations in preoperative pain and function, four included data from both US and European institutions [7, 15, 17, 18] and addressed either knee or hip patients. A more complete understanding of the similarities and differences in TKA and THA patients is required to guide risk-adjustment methods when making cross-cultural outcome comparisons.

Our objective was to describe and compare preoperative TKA and THA patient characteristics from two large prospective cohorts, one in Switzerland (CH) and the other in the United States (US). If significant differences in patient risk factors are detected, this will support the need for risk-adjustment prior to future international comparisons of outcomes.

## Methods

### Study participants and study design

Patient characteristics were collected prospectively on all elective primary TKAs and THAs performed (1) at a large University hospital in Geneva, Switzerland between January 2010 and December 2012 and (2) in FORCE-TJR, a diverse, national sample of more than 130 surgeons in the US between May 2011 and December 2012.

### Cohort from Switzerland

Swiss patients included in this study are part of two registries at the Division of Orthopaedic and Trauma Surgery of the Geneva University Hospitals, Geneva, Switzerland: (1) the THA registry prospectively enrolling all patients undergoing THA since 1996; and (2) the TKA registry prospectively enrolling all patients undergoing THA since 1998. The registries are approved by the local Ethics committee. The institution is one of the largest Orthopaedic and Trauma Surgery centers in Switzerland and the only public hospital of the Canton Geneva serving a population of 500,000 inhabitants. The institution treats patients with public insurance (about 85% of the patients operated upon for TJA) as well as those with private insurance (about 15%). Switzerland has universal compulsory health care coverage. The patients in the two registries were comparable in age and sex-distribution to the Swiss population undergoing hip or knee replacement [19–21].

### Cohort from the United States

FORCE-TJR (Function and Outcomes Research for Comparative Effectiveness in Total Joint Replacement) is a prospective, national total joint arthroplasty (TJA) cohort centered at the University of Massachusetts Medical School. Patients are enrolled from more than 130 orthopedic practices distributed across 22 states in the United States with diverse geography (e.g. urban/rural), ownership (e.g., HMO, private practice, and academic settings), and varied surgical volume to ensure inclusion of diverse patients and surgeon practices [22]. Participating surgeons identify consecutive TJA patients and the FORCE-TJR staff collect pre- and post-operative data. Patient demographic and clinical profiles parallel current US experience when compared to the most recent Healthcare Utilization Project (HCUP) data. FORCE-TJR obtained Institutional Review Boards (IRBs) approval, and patients provide informed consent. Participating surgeons also submit data detailing surgical implant details, surgical technique, operative data, hospitalization data, and patients report pain and functional outcome data at standard post-operative intervals. For simplicity, the FORCE-TJR cohort will be referred to as the US cohort.

### Patient characteristics, patient-reported outcome measures, and data collection

In Switzerland, information on baseline patient characteristics (exposure) was obtained preoperatively on: age at operation; sex; education level (years of schooling in three categories); Body Mass Index (BMI); diagnosis (primary or secondary osteoarthritis as defined by the surgeon); and medical co-morbidities (diabetes, cardiac disease and stroke) were routinely obtained from the anaesthesia report and discharge summary. In the US, these same data were recorded at the time surgery was scheduled based upon the patient-report and surgeon records. The US registry did not record secondary osteoarthritis.

Patient-reported outcome measures (PROMS; outcomes) were obtained by questionnaires sent to the patients 14 days (Swiss) and 1–2 months (US) prior to surgery. Geneva used a reduced form of the Western Ontario McMaster Universities (WOMAC) pain and function [23] and a global health questionnaire, the 12-item short-form health survey (SF-12). The US estimated the WOMAC pain and function scores from the routinely collected (hip disability and osteoarthritis outcome score (HOOS) and Knee injury and Osteoarthritis Outcome Score (KOOS) sub-scores [24] and the 36-item short-form health survey (SF-36) physical and mental component scores [25] were collected. Higher scores on both the WOMAC and SF indicate less pain and better function/health.

Data missingness was assessed in both cohorts. Complete data were available for 80% of patients in the Swiss cohort and 92% for the US. The one exception was education level because education was not assessed in the early years of the Swiss cohort, but was complete in the later years.

### Statistical analysis

We calculated differences in proportions and their 95% confidence intervals (CI) to compare categorical variables, and mean differences and 95% CIs to compare continuous variables. In addition, preoperative score differences were assessed using effect sizes. Effect sizes were calculated as mean unadjusted difference divided by the pooled SD of the corresponding mean scores. Effect sizes of 0.2, 0.5, and 0.8 are regarded as small, medium, and large degrees of difference, respectively [26]. For the WOMAC instrument an effect size ≥ 0.25 or effects > 6% of the maximal score have been considered as the minimal clinically important difference [27]. To illustrate variability in key measures, density plots were generated using STATA version 12 to compare US and Swiss distributions of age, BMI, and pre-TKA and THA pain and function. Scatter plots and lines of best fit display correlations between BMI and age, and pre-TKA and THA pain and function in the US and Switzerland as well as correlations between emotional health (Short Form health survey, Mental Component Summary; SF-MCS) and pre-TKA and THA pain and function. Pain and Function stratified by education were illustrated with bar graphs. Finally, we used linear regression with country as an indicator variable to assess differences by country in the associations between age, BMI, education, and MCS and pre-operative pain and function.

## Results

Overall, 6,680 primary TKAs from the US cohort and 823 primary TKAs from the Swiss cohort were included (see baseline characteristics: Table 1, categorical variables and Table 2, continuous variables), as well as 4,647 primary THAs from the US cohort and 1,023 primary THAs from the Swiss cohort (see baseline characteristics: Table 3, categorical variables and Table 4, continuous variables). Over the same time period, in the US registry more TKAs than THAs were implanted, which parallels relative TKA-THA utilization nationally, whereas in the Swiss cohort there were more THAs than TKAs.

**Table 1:** Baseline characteristics of patients undergoing primary TKA for categorical variables

	Geneva registry Switzerland (*N* = 823)	FORCE-TJR United States (*N* = 6,680)	Risk difference (95% CI)	*P*-value****
Women (N, %)	567 (68.9)	4,142 (62.0)	6.9 (3.5; 10.3)	0.000
Age in categories (N, %)				0.000
<60 years of age	75 (9.1)	1,462 (21.9)	-12.8 (-15.2; -10.5)	
60 to 79.9 years of age	555 (67.4)	4,623 (69.3)	-1.9 (-7.8; 4.1)	
≥80 years of age	193 (23.5)	586 (8.8)	14.7 (11.3; 18.1)	
Preoperative BMI categories (N, %)*				0.000
<25	177 (21.5)	832 (12.9)	8.6 (5.3; 11.9)	
25–29.9	295 (35.8)	2,053 (31.8)	4.0 (-0.3; 8.3)	
30–34.9	211 (25.6)	1,869 (29.0)	-3.3 (-7.0; 0.4)	
≥35	140 (17.0)	1,698 (26.3)	-9.3 (-12.4; -6.2)	
Education (N, %)**				0.000
<= 8 years	140 (41.1)	104 (1.6)	39.4 (32.6; 46.2)	
9–12 years (high school)	110 (32.3)	2060 (32.3)	0.0 (-6.2; 6.2)	
> = 13 years (college)	91 (26.7)	4216 (66.1)	-39.4 (-45.2; -33.6)	
Co-morbidities (N, %)				
Diabetes	145 (17.6)	1,125 (16.8)	0.8 (-2.3; 3.8)	0.932
Cardiac disease	48 (5.8)	531 (7.9)	-2.1 (-3.9; -0.3)	0.013
Stroke	24 (2.9)	209 (3.1)	-0.2 (-1.5; 1.0)	0.545
Primary OA (N, %)	719 (87.4)	5,886 (88.1)***	-0.7 (-3.2; 1.7)

**Table 2:** Baseline characteristics of patients undergoing primary TKA for continuous variables

	Geneva registry Switzerland (*N* = 823)	FORCE-TJR United States (*N* = 6,680)	Mean difference (95% CI)	*P*-value****
Age at operation, mean [±SD]	72.3 [±9.4]	66.7 [±9.4]	5.6 (4.9; 6.3)	0.000
Preoperative BMI, mean [±SD]*	29.6 [±5.9]	31.53 [±6.2]	-1.9 (-1.5; -2.4)	0.000
Mean age in each BMI category, [±SD]				
<25	72.9 [±10.9]	69.38 [±10.2]	3.5 (1.8; 5.2)	0.000
25–29.9	74.25 [±8.6]	68.51 [±9.4]	5.8 (4.6; 6.9)	0.000
30–34.9	71.02 [±8.8]	66.64 [±8.7]	4.4 (3.1; 5.6)	0.000
≥35	69.16 [±8.7]	63.29 [±8.5]	5.9 (4.4; 7.3)	0.000
Preoperative scores, mean [±SD]			Mean difference	
			(95% CI)/Effect size	
WOMAC pain	41.0 [±17.9]	51.59 [±19.0]	-10.6 (-12.2; -9.1)/0.57	0.000
WOMAC function	44.82 [±19.2]	52.09 [±18.6]	-7.3 (-8.9; -5.7)/0.39	0.000
SF-12 Physical Component Summary	34.45 [±7.6]	32.68 [±8.3]	1.8 (1.1; -2.5)/0.23	0.000
SF-12 Mental Component Summary	44.84 [±11.2]	51.40 [±12.3]	-6.6 (-7.6; -5.5)/0.56	0.000

*BMI was available for 6,452 TKAs (96.6%) from FORCE-TJR

**In the Geneva cohort information on education was routinely collected only since 2012

***FORCE-TJR collects RA versus OA

****Pearson chi2 tests were used for categorical variables, and two-sample Wilcoxon rank-sum (Mann-Whitney) tests were used for continuous variables

**Table 3:** Baseline characteristics of patients undergoing primary THA for categorical variables

	Geneva registry Switzerland (*N* = 1,023)	FORCE-TJR United States (*N* = 4,647)	Risk difference (95% CI)	*P*-value****
Women (N, %)	590 (57.7)	2,660 (57.2)	0.5 (-2.9; 3.8)	0.788
Age in categories (N, %)				0.000
<60 years of age	231 (22.6)	1,435 (31.0)	-8.4 (-11.8; -5.1)	
60 to 79.9 years of age	599 (58.6)	2,813 (60.8)	-2.2 (-7.4; 3.0)	
≥80 years of age	193 (18.9)	380 (8.2)	10.7 (7.9; 13.4)	
Preoperative BMI categories (N, %)*				0.000
<25	381 (37.5)	1,104 (24.3)	13.1 (9.1; 17.1)	
25–29.9	397 (39.0)	1,692 (37.3)	1.7 (-2.5; 6.0)	
30–34.9	173 (17.0)	1,056 (23.3)	-6.3 (-9.2; -3.4)	
≥35	66 (6.5)	683 (15.1)	-8.6 (-10.5; -6.6)	
Education (N, %)**				0.000
<= 8 years	126 (38.3)	36 (0.8)	37.5 (30.8; 44.2)	
9–12 years (high school)	104 (31.6)	1134 (25.7)	5.9 (-0.4; 12.1)	
> = 13 years (college)	99 (30.1)	3238 (73.5)	-43.4 (-49.8; -36.9)	
Co-morbidities (N, %)				
Diabetes	98 (9.6)	543 (11.7)	-2.1 (-4.2; 0.0)	0.017
Cardiac disease	80 (7.8)	330 (7.1)	0.7 (-1.2; 2.6)	0.610
Stroke	38 (3.7)	116 (2.5)	1.2 (0.0; 2.5)	0.050
Primary OA (N, %)	834 (81.5)	4,190 (90.2)***	-8.7 (-11.2; -6.1)

**Table 4:** Baseline characteristics of patients undergoing primary THA for continuous variables

	Geneva registry Switzerland (*N* = 1,023)	FORCE-TJR United States (*N* = 4,647)	Mean difference (95% CI)	*P*-value****
Age at operation, mean [±SD]	68.2 [±12.9]	64.6 [±10.7]	3.6 (2.8; 4.3)	0.000
Preoperative BMI, mean [±SD]*	26.87 [±5.0]	29.12 [±5.7]	-2.2 (-1.9; -2.6)	0.000
Mean age in each BMI category, [±SD]				
<25	68.61 [±14.7]	66.35 [±11.5]	2.3 (3.7; 0.8)	0.000
25–29.9	68.55 [±12.2]	65.42 [±10.4]	3.1 (1.9; 4.3)	0.000
30–34.9	67.24 [±11.0]	63.82 [±10.1]	3.4 (1.8; 5.1)	0.000
≥35	65.68 [±8.8]	61.17 [±9.8]	4.5 (2.1; 7.0)	0.000
Preoperative scores, mean [±SD]			Mean difference	
			(95% CI)/Effect size	
WOMAC pain	39.51 [±18.5]	47.81 [±19.8]	-8.3 (-9.8; -6.8)/0.43	0.000
WOMAC function	40.21 [±19.5]	44.24 [±19.2]	-4.0 (-5.6; -2.5)/0.21	0.000
SF-12 Physical Component Summary	33.39 [±7.9]	31.26 [±8.5]	2.1 (1.5; 2.8)/0.26	0.000
SF-12 Mental Component Summary	43.91 [+11.7]	49.97 [±12.6]	-6.1 (-7.0; -5.1)/0.5	0.000

*BMI was available for 6,452 TKAs (96.6%) from FORCE-TJR

**In the Geneva cohort information on education was routinely collected only since 2012

***FORCE-TJR collects RA versus OA

****Pearson chi2 tests were used for categorical variables, and two-sample Wilcoxon rank-sum (Mann-Whitney) tests were used for continuous variables

### Between-cohort comparison of baseline characteristics prior to knee arthroplasty

Patients undergoing primary TKA in the US compared to those in Switzerland were significantly younger (mean age 67 vs. 72 years.; <60 years: 22% vs. 9%, p < .0001). The mean BMI differed between the two registries (31.5 vs. 29.6 kg/m^2^) with the US having significantly more obese class I (BMI 30-34.9: 29% vs. 26%, p < .0001) and class II patients (BMI ≥35: 26% vs. 17%, p < .0001). Patients in the US reported cardiac disease more often, despite a younger mean age. With respect to preoperative scores, patients in the US had higher WOMAC pain scores (52 vs. 41 points) indicating significant knee pain in both countries, but less knee-specific pain at time of TKA in the US. The effect size was clinically relevant (0.57). While WOMAC function scores in both countries indicated significant impairment, knee-specific function scores were higher in the US (52.1 vs. 44.8). Pre-operative overall physical disability as measured by the general health questionnaire (SF) was reported in both countries, (Swiss 34.5 vs. US 32.7). The mental component score of the SF was lower in the Swiss cohort (effect size 0.56).

### Between-cohort comparison of baseline characteristics prior to hip arthroplasty

Patients undergoing primary THA in the US compared to those in Switzerland were significantly younger (mean age 65 vs. 68 years.; <60 years: 31% vs. 23%, p < .0001). The mean BMI differed between the two registries (29.1 vs. 26.9 kg/m^2^) with the US having more obese class I (BMI 30-34.9: 23% vs. 17%, p < .0001) and class II patients (BMI ≥35: 15% vs. 7%, p < .0001). Patients in the US had also more often diabetes, despite a younger mean age. With respect to preoperative scores, patients in the US had higher WOMAC pain scores (48 vs. 40 points) indicating less hip-specific pain at time of THA. The effect size was clinically relevant (0.43). WOMAC function scores were also higher in the US indicating slightly less functional impairment due to hip OA. Marked pre-operative overall physical disability on the general health questionnaire (SF) was reported in both countries,(US 31.3 vs. Swiss 33.4). The mental component score of the SF was lower in the Swiss cohort (effect size 0.50).

### Between-cohort variation in age, BMI, education, MCS, and pre-operative pain and function

**Fig. 1:**
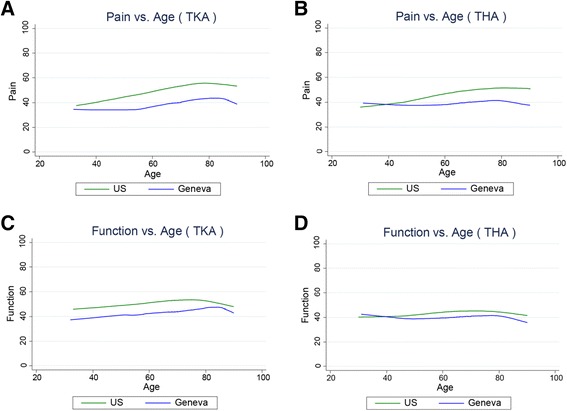
Association between Age and pre-THA/TKA pain/function among US (FORCE) and Swiss (Geneva) patients. Lower scores represent greater pain/poorer function. **a**. association pre-TKA WOMAC pain; **b**. pre-THA WOMAC pain; **c**. pre-TKA WOMAC function; **d**. pre-THA WOMAC function. The association between pain and age in (**b**). (THA) is significantly different between countries (P < 0.002)

**Fig. 2:**
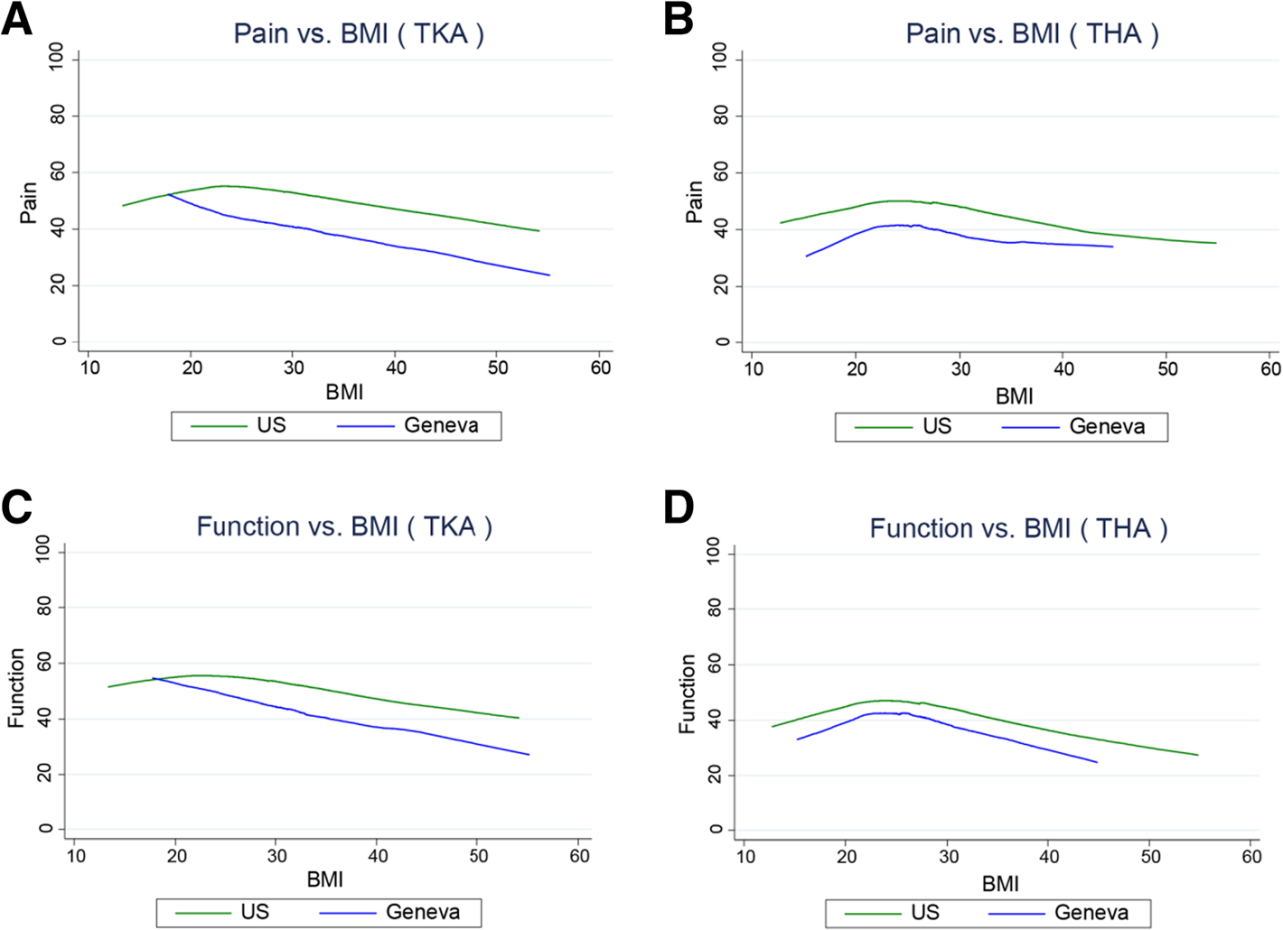
Association between BMI and and pre-THA/TKA pain/function among US (FORCE) and Swiss (Geneva) patients. Lower scores represent greater pain/poorer function. **a**. association pre-TKA WOMAC pain; **b**. pre-THA WOMAC pain; **c**. pre-TKA WOMAC function; **d**. pre-THA WOMAC function. The association between function and BMI in (**c**). (TKA) is significantly different between countries (P < 0.046)

**Fig. 3:**
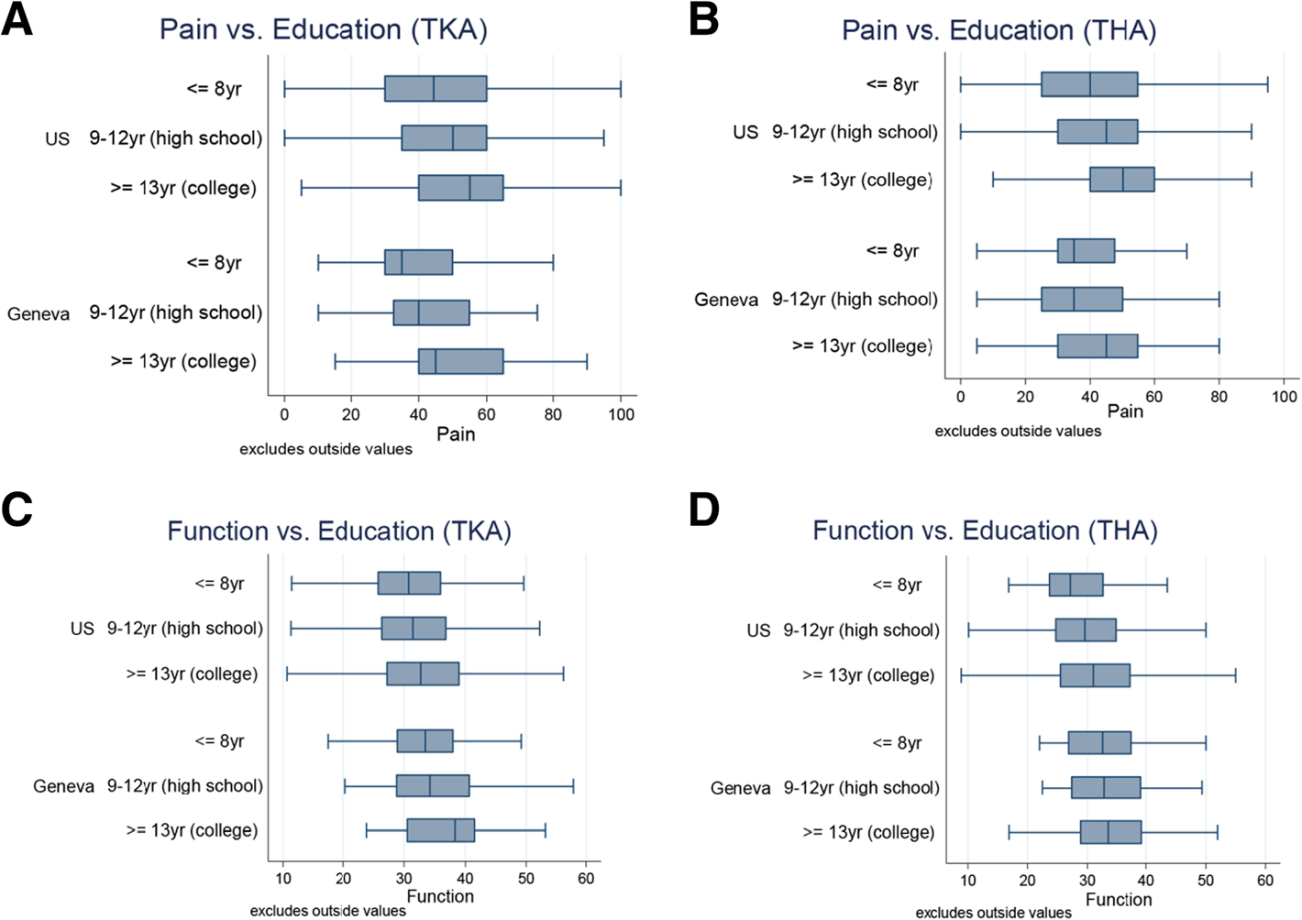
Association between Education and pre-THA/TKA pain/function among US (FORCE) and Swiss (Geneva) patients. Lower scores represent greater pain/poorer function. **a**. association pre-TKA WOMAC pain; **b**. pre-THA WOMAC pain; **c**. pre-TKA WOMAC function; **d**. pre-THA WOMAC function. No statisticial difference between countries was detected for TKA or THA in the association between education and pain and function

**Fig. 4:**
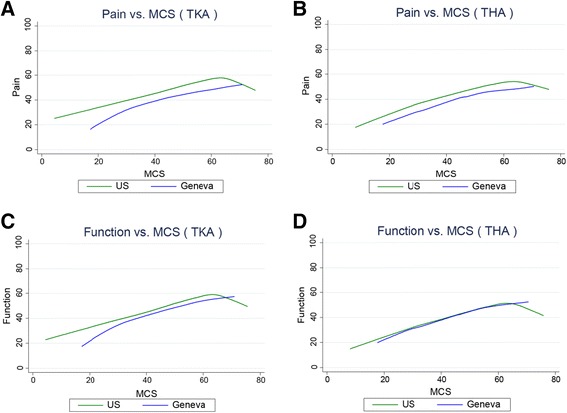
Association between emotional health (SFMCS) and pre-THA/TKA pain/function among US (FORCE) and Swiss (Geneva) patients. Lower scores represent greater pain/poorer emotional health. **a**. association pre-TKA WOMAC pain; **b**. pre-THA WOMAC pain; **c**. pre-TKA WOMAC function; **d**. pre-THA WOMAC function. No statisticial difference between countries was detected for TKA or THA in the association between education and pain and function

Analyses of the variation in age, BMI, education, MCS, and pre-operative pain and function found that similar variation exists in both cohorts on each measure. (Figures 1, 2, 3 and 4). However, the Swiss distributions are shifted toward older age, lower BMI, lower education level, lower MCS, and lower pain score (greater pain) for both TKA and THA. In contrast, the pre-operative function score distributions are similar in both countries for TKA and THA. In both TKA and THA, mean BMI was higher in US patients than in Swiss patients across all age groups. Within each BMI category, US TKA and THA patients were substantially younger as compared to the Geneva patients and the difference in BMI was greatest in the youngest patients. (Tables 1, 2, 3 and 4).

### Within-cohort correlations between age and pain and function

Similar relationships existed between age and preoperative pain and function for both TKA and THA. In TKA, older age in both Swiss and US patients was associated with less pre-operative pain and poorer function (lower WOMAC scores) (Fig. 1a and c). For THA patients, US patients with older age had less pain, but Swiss patients reported similar pain across ages (Fig. 1b). THA function was similar across all ages in both the US and Swiss patients (Fig. 1d). No statistical difference was detected between countries in the association between age and pain and function for TKA. However, in THA, the association with pain (not function) was significantly different (p < 0.002) between countries.

### Within-cohort correlations between BMI and pain and function

In both the US and Swiss patients, higher BMI was associated with greater pre-operative pain and poorer function (lower WOMAC scores) for both TKA and THA (Fig. 2a-d). No statistical difference was detected between countries in the association between BMI and pain and function for THA. However, in TKA, the association with function (not pain) was significantly different (p < 0.05) between countries.

### Within-cohort correlations between education and pain and function

In both the US and Swiss patients, lower education was associated with greater pre-operative pain (lower WOMAC scores) for both TKA and THA (Fig. 3a-d). Patients with less education also tended to have lower function, however the association was much weaker than for pain. No statisticial difference between countries was detected for TKA or THA in the association between education and pain and function.

### Within-cohort correlations between emotional health and pain and function

In both the US and Swiss patients, poorer emotional health (low MCS) was correlated with greater pre-operative pain and poorer function (Fig. 4a-d). This relationship was similar for both TKA and THA patients. No statistical difference between countries was detected for TKA or THA in the association between emotional health and pain and function.

## Discussion

At the time of primary total knee and hip arthroplasty, clinically important differences in age, education level, obesity prevalence, medical comorbidities, preoperative pain levels and emotional health were observed between the US and Swiss cohorts. The level of functional impairment at the time of TKA and THA reflected significant, and similar, disability in both countries. Because existing research documents that implant longevity, post-operative complication rates, and improvement in pain and function after TJA vary by patient characteristics, these data suggest that future cross-national TJA outcome comparisons should address pre-TJA patient differences prior to drawing conclusions [6–14].

Obesity is a well-known and important risk factor for short- and long-term complications and a threat to prosthesis survival [8, 13]. The pattern and prevalence of population obesity differs between the US and Switzerland, especially among TKA patients, and the differences have become more pronounced during the past decade. Between 1999 to 2009, the “Bus Santé”, a large cross-sectional population-based study in Geneva, reported 35% of the population were overweight and 12% obese [28]. The prevalence did not change during the 10 year time period [29]. In contrast, the corresponding prevalences of overweight and obesity in the US were 34% and 30.5%, respectively, in 1999, and 33% and 35.7%, in 2009 [30]. The observed prevalences of obesity among our TJA patients exceeded population-based prevalences, both in Geneva and in the US. The differences were larger in TKA as compared with THA patients. This finding parallels the understanding that obesity is an important risk factor for OA, especially for knee OA [31].

Obesity is also known to be associated with younger age at the time TJA is performed [32]. We found that the US TJA patients were substantially younger in every BMI category as compared to the Geneva patients, although the difference in age was highest in the greatest BMI category. Thus, increased BMI may explain only part of the observed age difference as reported previously [33, 34]. Other reasons for the younger age of the US TJA patients may be related to cultural expectations or health care access and delivery differences. Age is an important factor influencing joint replacement outcomes. Younger age increases the risk of prosthesis failure due to the generally more active lifestyle in younger people [35]. Because of the substantially younger age in the TKA and THA US cohorts there is an expected greater proportion of patients still working and thus likely exposing their arthroplasty to higher demands.

While patients in the US had greater BMI, the mean pain score among TJA patients in the US was less than the pain score reported in Switzerland. However, prior research has documented that greater BMI is associated with greater arthritis pain [36, 37]. This relationship exists in these data as well. In both the US and Swiss TKA and THA patients, higher BMI correlated with more pain. TJA patients in both countries reported similar, and substantial, functional limitations. Consistent with previous reports, higher BMI was associated with poorer function in both countries.

Only one study evaluated the association between education and preoperative pain and function levels [15] prior to TKA using the WOMAC score. In contrast to our results they did not report an association. However, Keurentjes et al. [38] using the SF-36 found lower preoperative scores in less educated THA and TKA patients. The discrepancy in education levels between the US and Swiss cohorts may be exaggerated in this study. The well-documented disparity in the use of TJA in the US among minority patients [39–45] and those with lower income and education will skew the education level in this cohort toward a higher mean US education [46]. In contrast, the proportion of patients with tertiary education in the Swiss cohort was in accordance with the reported levels of education in 2012 in Switzerland (29% with tertiary education) based on the annual OECD survey of adults aged 55–64 years [47]. However, of importance, both cohorts illustrate a significant association between lower education level and greater pre-operative pain.

Despite the younger age in US patients, the prevalence of comorbidities (diabetes- TKA, cardiac disease-THA) was greater. Diabetes and cardiac disease are both associated with obesity and with increased short-term post-TJA complication rates [9, 11, 14]. However, researchers have reported that medical comorbidities, in contrast to other patient factors, are not key predictors of patient-reported outcomes in THA [48]. In contrast to the pattern in medical comorbidities, emotional health, as measured by the SF MCS, was substantially poorer in Switzerland than in the US. However, a number of reasons may explain this difference. First, population-based normative values for the SF-12 MCS vary between the two countries (US MCS norm 50 as compared to the French-speaking area of Switzerland MCS norm 46.3, [49] accounting for some of this difference. Second, the poorer emotional health is possibly related to the greater pain level among the Swiss patients, because higher levels of OA pain have been associated with greater disability and depressed mood [50]. And third, patients with lower socioecomonic status, who constitute a much higher proportion in the Swiss as compared to the US cohort, have been shown to report lower emotional health [51]. The MCS differences are important as it has been reported that patients with poorer pre-TJA emotional health may be at risk for suboptimal postoperative outcomes [7]. A multicomponent psychosocial support program has been suggested prior to and following surgery including consistent counselor support as well as education and coping skills training to address anxiety, pain management, depression and the role of social supports [42].

In summary, the greater prevalence of obesity and medical comorbidities, plus a younger mean age, potentially increase the risk for complications and revisions among the US patients, as compared to the Swiss. However, the Swiss reported a much higher proportion of patients with a low level of education, higher pain levels and poorer emotional health at the time of surgery. These differences should be considered in future cross-cultural comparisons of short and long-term outcomes after TJA.

Few prior studies have evaluated differences in patient risk factors across international patient groups. In 2004, Lingard and colleagues evaluated the predictors of pain relief and functional gain after TKA in the US, England, and Australia [7]. While the researchers evaluated the role of BMI, only pre-operative physical function, poor emotional health, and greater medical comorbidities were associated with poorer outcomes. In a study of 12 European countries with nearly all THA patients having advanced radiographic hip OA, the level of pre-operative pain, disability, and patient risks varied across countries [44]. Another sample of TJA patients from 10 countries found TJA patients had worse mean pain and function scores than OA patients without TJA but there was substantial overlap in symptoms between the two groups, and no consistent pain and function profile existed for TJA patients across countries [17]. Gromov and colleagues reported that US THA patients had a younger age and higher BMI in accordance with our findings, however, they found greater pain and poorer function than in the European patients [18]. Finally, Gordon and colleagues report that patient predictors of pain and function in THA performed similarly across two countries (Denmark and Sweden) [52].

Surgeons, policy makers, and implant manufacturers rely on national registry reports for comparative implant information. Registry reports as those from the Scandinavian countries, the United Kingdom, or Australia present implant survival within age and sex sub-groups, and identify differences in implant survival by these patient attributes, While the United Kingdom, Sweden, the Netherlands, and New Zealand implant registries are now collecting PROMs [49–51, 53, 54] national registries do not yet adjust implant survival comparisons by pre-operative function and/or comorbidities. Our data document important differences in patient characteristics between TKA and THA patients from different countries. Future research will examine the impact of these differences on TJA outcomes.

### Limitations

While we carefully pre-defined measures to assure comparable data, there are possible measurement limitations. First, both countries are dependent upon documentation practices for medical comorbidities and it is possible that the difference between the US and Switzerland is related to differences in documentation. For example, the US database lacked documentation of secondary OA, while the Swiss registry was able to differentiate primary and secondary OA. Second, different methods were used to collect and score patient-reported outcomes (SF12 vs SF36; WOMAC vs HOOS/KOOS). However, previous psychometric research documents that the scores are comparable within SF versions and between the WOMAC and the KOOS. Finally, while this report is based on a sample of patients from the US and Switzerland, the respective registries have documented that the demographics of participating patients are comparable to the total population with health care coverage in these countries/regions and both cohorts include diverse surgeons.

## Conclusion

Clinically important differences in BMI, age at surgery, medical comorbidities, and preoperative pain and emotional health as well as differences in education level were observed between the US and Swiss cohorts before primary total knee and hip arthroplasty. The level of functional impairment at the time of TKA and THA reflected significant disability in both countries. The observed differences may result in differences in clinical outcomes, and complication and revision rates. Cross-cultural comparisons of TJA outcomes should consider risk-adjustment for these variables prior to making conclusions about apparent differences in outcome. Future studies will evaluate post-TJA outcomes and test the role of risk-adjustment prior to making these comparisons.

## Abbreviations

BMI: Body mass index
FORCE-TJR: Function and outcomes research for comparative effectiveness in total joint replacement
HCUP: Healthcare utilization project
HOOS: Hip disability and osteoarthritis outcome score
IRBs: Institutional Review Boards
KOOS: Knee injury and osteoarthritis outcome score
OA: Osteoarthritis
PROMs: Patient-reported outcome measures
SF-36: 36-item short-form health survey
SF-MCS: Short form health survey, mental component summary
THA: Total hip arthroplasty
TJA: Total joint arthroplasty
TKA: Total knee arthroplasty
US: United States
WOMAC: Western Ontario McMaster Universities
